# Evaluating a Coenzyme Q10-Based Food for Special Medical Purpose, for Mitochondrial Diseases Management: An Open-Label, Pilot Trial

**DOI:** 10.3390/ijms27115127

**Published:** 2026-06-05

**Authors:** Lucia Chico, Piervito Lopriore, Giulia Cecchi, Adriana Meli, Clara Bernardini, Linda Balestrini, Maico Polzella, Vincenzo Montano, Michelangelo Mancuso

**Affiliations:** 1 Laboratori Aliveda S.r.l., Crespina Lorenzana, 56042 Pisa, Italy; l.balestrini@laboratorialiveda.com (L.B.); maico@aliveda.com (M.P.); 2 Department of Clinical and Experimental Medicine, Neurological Institute, University of Pisa, 56100 Pisa, Italy; piervitolopriore@gmail.com (P.L.); giuliacecchi19@gmail.com (G.C.); adrianameli97@gmail.com (A.M.); c.bernardini96@gmail.com (C.B.); v.montano89@gmail.com (V.M.); michelangelo.mancuso@unipi.it (M.M.); 3 Ph.D. School in Translational Medicine, Scuola Superiore Sant’Anna, 56127 Pisa, Italy; 4 CTMM—Interdepartmental Research Centre for Translational Medicine in Neuromuscular and Mitochondrial Diseases, University of Pisa, 56100 Pisa, Italy

**Keywords:** mitochondrial diseases, coenzyme Q10, food for special medical purposes, antioxidants

## Abstract

Primary mitochondrial diseases (PMD) are rare disorders with limited therapeutic options. Coenzyme Q10 (CoQ10) supplementation is widely used, although formulation differences can affect absorption and efficacy. This open-label pilot feasibility trial evaluated a food for special medical purposes (FSMP) containing high-dose CoQ10 (250 mg per capsule) in patients with PMD. Ten patients (mean age: 55.5 ± 8.6 years) were enrolled. Serum/plasma biomarkers, including CoQ10, fibroblast growth factor 21 (FGF21), growth differentiation factor 15 (GDF15), ferric-reducing antioxidant power (FRAP), total sulfhydryl groups (t-SH), and advanced oxidation protein products (AOPP), were assessed at baseline (T0, after ≥30 days of conventional ubidecarenone) and after 30 days of FSMP administration (T1). Fatigue severity scale (FSS) and 5-times sit-to-stand test (5xSST) were evaluated at both timepoints. FSMP was administered at 250 or 500 mg/day. Twenty sex- and age-matched healthy controls were included for CoQ10 comparison. Absolute CoQ10 concentrations remained stable overall at T1, with all patients maintaining levels above 390 ng/mL (100% vs. 60% at T0), although concentrations remained lower than in healthy controls (*p* < 0.01). Dose-normalized CoQ10 exposure was significantly higher with FSMP versus conventional ubidecarenone (*p* < 0.001, Cohen’s d = 7.31). FGF21, GDF15, AOPP, and t-SH remained unchanged, whereas FRAP increased at T1 (*p* < 0.01). No significant changes were observed in 5xSST and FSS. Exploratory analyses indicated inter-individual variability in functional responses. FSMP was associated with higher dose-normalized systemic CoQ10 exposure, more consistent circulating CoQ10, and increased FRAP levels. Its simplified dosing regimen may support long-term adherence. Larger studies are warranted to validate these preliminary findings.

## 1. Introduction

Primary mitochondrial diseases (PMD) comprise a heterogeneous group of disorders characterized by impaired mitochondrial function. Mitochondria generate ATP through oxidative phosphorylation (OXPHOS), a process driven by the electron transport chain (ETC, complexes I–IV) and ATP synthase (complex V). Disruption to any ETC or OXPHOS system compromise ATP production, resulting in mitochondrial dysfunction and cellular bioenergetic failure [1].

PMD arise from pathogenic variants in either mitochondrial DNA (mtDNA) or nuclear DNA (nDNA), affecting not only OXPHOS but also proteins involved in mtDNA replication and maintenance, mitochondrial protein import, and mitochondrial dynamics [2].

Mitochondria are ubiquitous organelles that play a central role in cellular metabolism; therefore, mitochondrial dysfunction can affect virtually any organ system. PMD are clinically heterogeneous and may manifest at any age. They can be tissue-specific or multi-systemic, and organs with high energy demands, such as the brain, skeletal muscle, eyes, and heart, are particularly vulnerable to energy deficiency [3].

In response to mtDNA defects, mitochondrial stress triggers a gradual cellular response that increases the production of stress-responsive mitokines, including fibroblast growth factor 21 (FGF21) and growth differentiation factor 15 (GDF15). FGF21 and GDF15 are key regulators of energy balance and glucose homeostasis, and their evaluation has been proposed as a hallmark of mitochondrial stress [4,5].

In addition to impaired ATP synthesis, mitochondrial disorders are characterized by redox imbalance, including increased oxidative damage and defective antioxidant defenses. These alterations further exacerbate damage to mtDNA, nDNA, proteins, and lipids, ultimately driving cellular degeneration [6]. Given these mechanisms, some current therapeutic approaches focus on mitigating oxidative stress [7]. Among antioxidant agents, coenzyme Q10 (CoQ10)—or ubiquinone—has received particular attention for its safety profile and its dual role as an electron carrier within the ETC and as a lipid-soluble antioxidant [8]. Nevertheless, current clinical evidence does not support a generalized benefit of CoQ10 supplementation across the mitochondrial disease spectrum [9,10]. Notably, specific genetic subgroups, such as primary CoQ10 deficiency (PCoQ10D) and the carriers of the m.10197 G > A mutation in the *ND3* gene, can respond to CoQ10 treatment, particularly with respect to non-central nervous system manifestations, including fatigue, exercise intolerance, peripheral neuropathy, and, in some cases, ataxia [11,12,13]. Furthermore, idebenone, a synthetic CoQ10 analogue, has been approved by the European Medicines Agency (EMA) for the treatment of Leber Hereditary Optic Neuropathy (LHON), with clinical use primarily supported in acute or dynamic disease stages [14,15]. Despite these premises, the clinical efficacy of CoQ10-based compounds is often limited by poor intestinal absorption and marked inter-individual variability in bioavailability, potentially reducing therapeutic effectiveness [16]. In this context, strategies aimed at improving systemic availability remain of interest. Notably, a recent first-in-human therapeutic trial using 4-hydroxybenzoic acid, a biosynthetic precursor of CoQ10, in a child with *CoQ2* deficiency suggested that enhancing coQ10-related metabolic availability may translate into clinical benefit, underscoring the potential relevance of formulation- or pathway-based approaches [17].

In Italy, the most commercially available CoQ10-containing products provide between 30 mg and 200 mg representing the highest dose allowed for general use in food supplements. As a result, achieving higher therapeutic doses typically requires multiple daily administration, increasing pill burden and potentially reducing treatment adherence.

To address these limitations, the efficacy of a high-dose CoQ10-based formulation provided as a food for special medical purpose (FSMP) was evaluated in adult patients with PMD. Specifically, the study assessed whether high-dose CoQ10 supplementation was associated with improvements in functional performance, fatigue severity, and biochemical markers of mitochondrial damage and oxidative stress.

## 2. Results

Ten patients with genetically confirmed mitochondrial disease (50% male) were enrolled. Baseline demographic and clinical characteristics are reported in Table 1 and Table S1. The mean age was 55.5 ± 8.7 years and the mean body mass index (BMI) was 21.1 ± 4.0 kg/m^2^. The mean age at disease onset was 39 ± 19.8 years (range 11–68), and mean disease duration was 145.3 ± 180.3 months. Prior to enrollment, patients had received ubidecarenone for a mean of 88.7 ± 72.4 months; dosing regimens for conventional CoQ10 formulations and the FSMP are reported in Table 1. Twenty healthy controls (45% male) were included in the CoQ10 analysis. Controls had a mean age of 48.0 ± 7.2 years and a mean BMI of 24.9 ± 3.8 kg/m^2^, which was significantly higher than that of patients (*p* = 0.02). Among controls, 50% were of normal weight, 35% were overweight, and 15% were obese. Additional information on mtDNA mutations, muscle manifestations, and the type of conventional formulation used is provided in Table S1.

**Figure 1:**
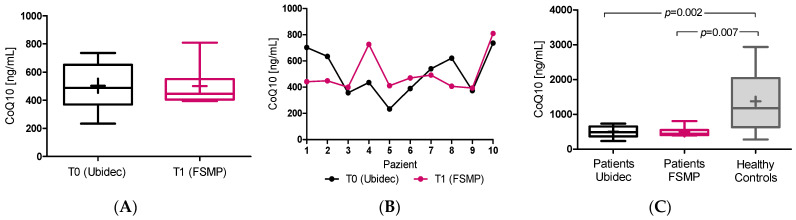
Serum CoQ10 concentrations and subgroup comparisons. (**A**) Box-plots (median; IQR; min–max range; +: mean value); (**B**) individual patient changes; (**C**) comparison between patients (Ubidecarenone and FSMP subgroups) and healthy controls. Ubidec: ubidecarenone, FSMP: food for special medical purposes.

Serum CoQ10 concentrations did not change significantly between T0 and T1 (Figure 1A). Individual trajectories were heterogeneous: 6/10 patients showed increased levels, whereas 4/10 patients exhibited a decrease after switching to the FSMP (Figure 1B). At T1, all patients showed serum CoQ10 concentrations above 390 ng/mL (range: 394.1–809.6 ng/mL), corresponding to the lowest value observed following FSMP administration; by contrast, only 60% of patients reached this level during conventional ubidecarenone treatment at T0 (range: 233.5–736.6 ng/mL) (Figure 1A,B). Despite supplementation, CoQ10 levels remained significantly lower in patients compared with healthy controls at both T0 (*p* = 0.002) and T1 (*p* = 0.007) (Figure 1C).

**Figure 2:**
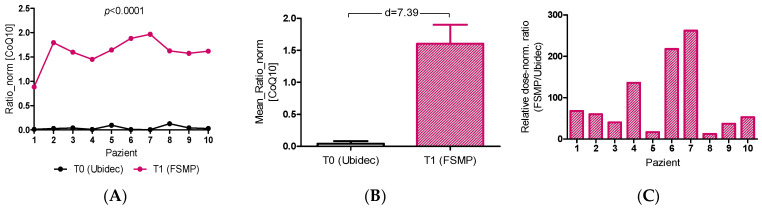
Dose-normalized CoQ10 Ratio (Ratio_norm). (**A**) Individual patient values at T0 and T1; (**B**) group mean at T0 and T1; (**C**) relative dose-normalized exposure ratio (FSMP/Ubidecarenone). T0: conventional ubidecarenone; T1: FSMP.

When normalized for cumulative dose, dose-normalized CoQ10 ratio (Ratio_norm) was significantly higher with FSMP compared to conventional ubidecarenone (*p* < 0.001; Figure 2A). The effect size calculated on mean Ratio_norm values confirmed markedly greater absorption efficacy for FSMP (Cohen’s d = 7.39; Figure 2B). The relative dose-normalized exposure ratio (FSMP/Ubidecarenone) demonstrated FSMP superiority across all patients (median: 56.4-fold; range: 12.6–262.2), with substantial inter-individual variability observed (Figure 2C).

Moreover, no significant correlation was observed between CoQ10 levels and BMI in patients at either T0 or T1 (Supplementary Materials, Figure S1A), nor in healthy controls (Supplementary Materials, Figure S1B). Similarly, no significant associations were detected when CoQ10 normalized absorption and relative bioavailability were considered (Supplementary Materials, Table S2). In BMI-adjusted sensitivity analyses, the between-group difference in circulating CoQ10 remained significant (*p* = 0.007), whereas BMI was not associated with CoQ10 concentrations (*p* = 0.971) (Supplementary Materials, Table S3).

**Figure 3:**
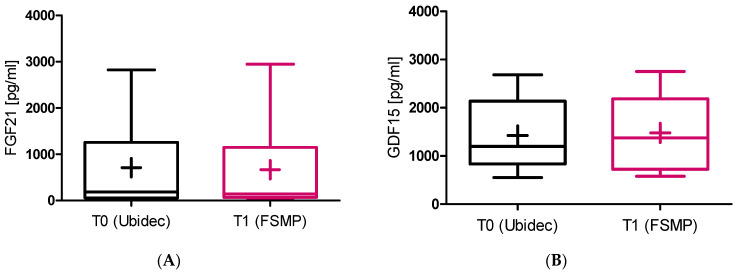
Mitokine levels at T0 and T1. (**A**) Fibroblast growth factor 21 (FGF21); (**B**) growth differentiation factor 15 (GDF15). The symbol “+” indicates the mean.

Mitochondrial stress biomarkers FGF21 (Figure 3A) and GDF15 (Figure 3B) did not show significant changes between T0 and T1.

FRAP levels increased significantly at T1 (1.141 ± 0.29 mmol/L) compared to T0 (0.954 ± 0.33 mmol/L) (*p* < 0.01) (Supplementary Materials, Figure S2A). In contrast, no significant changes were observed in t-SH levels (Supplementary Materials, Figure S2B) and AOPP levels (Supplementary Materials, Figure S2C).

**Figure 4:**
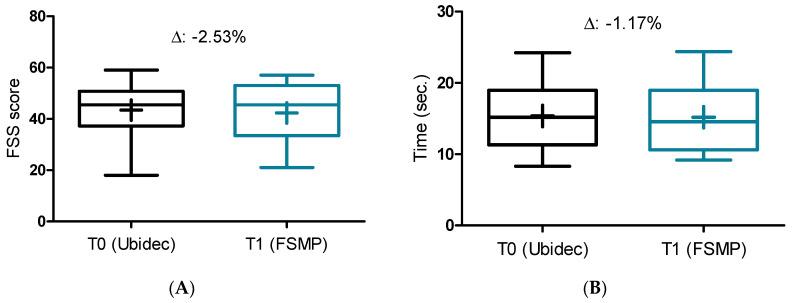
Clinical outcomes at T0 and T1. (**A**) Fatigue severity scale, FSS; (**B**) 5-time sit to stand test, 5xSST; mean percentage change, Δ (%): T1−T0. The symbol “+” indicates the mean.

**Figure 5:**
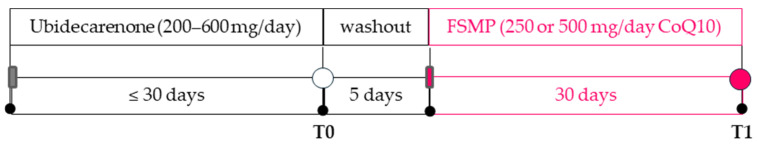
Study design overview. After baseline assessment (T0), patients underwent a 5-day washout from conventional ubidecarenone supplementation (200–600 mg/day), followed by 30 days of FSMP administration (250 or 500 mg CoQ10/day) with assessment at T1.

Regarding clinical outcomes, FSS (Figure 4A) and 5xSST (Figure 4B) showed no significant changes between T0 and T1. Mean percentage changes were −2.53% for FSS and −1.17% for 5xSS.

No treatment discontinuations, serious adverse events, or clinically relevant gastrointestinal adverse effects were observed during the 30-day supplementation period.

### Exploratory Subgroup Analyses

Given the substantial inter-individual variability observed in the primary analyses, exploratory subgroup analyses were performed. Owing to the small sample size, these findings are descriptive and hypothesis-generating; therefore, no formal inferential comparisons between subgroups were conducted.

Patients were classified into two groups based on predominant clinical phenotype (Table 2):

- Point mutation with multisystem involvement (*n* = 4), including MELAS, MIDD, and NARP;
- Myopathy (*n* = 6), including PMM with or without PEO, single mtDNA deletion-associated conditions, and KSS spectrum.

Four patients (AL01, AL02, AL07, and AL08) showed a reduction in serum CoQ10 following formulation switch (ΔCoQ10: −261.1, −185.9, −48.5, and −214.8 ng/mL, respectively) (Table 2). All four had high baseline CoQ10 concentrations (range: 540.5–703.2 ng/mL and had received conventional ubidecarenone for a mean duration of 80.5 months prior to the switch (Table 1). Despite the reduction in circulating CoQ10, three of these four patients (AL01, AL07, AL08) improved in at least one functional outcome at T1, and all four showed increased FRAP levels (mean ΔFRAP: +0.275 mmol/L) (Table 2).

In this exploratory subgroup analyses, mean serum CoQ10 increased in the point mutation/multisystem group (ΔCoQ10: +62.5 ng/mL; 3/4 patients showed an increase) and decreased in the myopathy group (ΔCoQ10: −51.8 ng/mL; 3/6 patients showed an increase). Despite the reduction in circulating CoQ10, the myopathy group also showed numerically favorable changes in functional measures (mean Δ5xSST: −0.99 s; mean ΔFSS: −4.5 points) and a greater improvement in FRAP (mean ΔFRAP: +0.208 mmol/L) compared with the point mutation/multisystem group (mean Δ5xSST: +1.02 s; mean ΔFSS: +4.0 points; mean ΔFRAP: +0.154) (Table 3).

Individual ΔCoQ10 values and their relationship with disease duration are shown in Supplementary Figure S3A and S3B, respectively.

Individual percentage changes (Δ%) in serum CoQ10 levels, 5xSST, and FSS, are reported in Supplementary Table S4.

In addition, as a complement to the main analysis, a responder analysis was performed to descriptively summarize the proportion of patients showing predefined, potentially clinically relevant changes in functional endpoints. For FSS, 60% of patients achieved a reduction of ≥2 points; using more conservative thresholds (≥3 and ≥4 points), the proportion of responders was 40% and 30%, respectively. For the 5xSST, 20% of patients showed a reduction in execution time >2 s (Supplementary Table S5).

## 3. Discussion

Despite substantial progress in molecular and biochemical diagnostics, effective therapeutic strategies remain limited. Current management, therefore, mainly relies on supportive interventions aimed at enhancing mitochondrial function or mitigating the consequences of impaired oxidative phosphorylation, with CoQ10 among the most frequently used supplements [20].

A broad spectrum of conditions associated with reduced CoQ10 availability–including mitochondrial diseases–may benefit from CoQ10 supplementation [21]. In PCoQ10D, early high-dose oral therapy has shown clinical benefit [21], whereas responses in secondary CoQ10 deficiencies are often variable or incomplete, likely reflecting the multifactorial mechanisms underlying impaired CoQ10 homeostasis [22]. In this context, formulation characteristics are particularly relevant, as systemic exposure appears to be influenced less by the redox form—ubiquinone versus ubiquinol—than by the delivery matrix and absorption efficiency [23]. Marked variability in CoQ10 absorption across formulations and among individuals has been reported in heterogeneous populations [24,25], highlighting the need for strategies that improve exposure consistency in PMD, where metabolic demands are substantially increased [26].

In this study, adult patients with genetically confirmed PMD transitioned from conventional ubidecarenone to a novel high-dose CoQ10 formulation (FSMP, Q250^®^), designed to simplify administration while maintaining CoQ10 delivery.

Absolute serum CoQ10 concentrations did not differ significantly between conventional ubidecarenone and FSMP treatment. However, individual trajectories revealed variability: six patients showed increased CoQ10 levels, whereas four exhibited a decrease after switching to FSMP. Notably, under FSMP all patients maintained serum concentrations above the lowest value observed after FSMP administration (394 ng/mL), whereas this threshold was reached by only 60% of patients during conventional treatment. Although descriptive and not clinically validated, this pattern is consistent with a more homogeneous exposure profile with FSMP. Post-FSMP concentrations also overlapped with the upper range previously reported for high-bioavailability dry-powder CoQ10 formulations (0.15–0.45 μg/mL) [27].

Nevertheless, serum concentrations remained constantly lower than those observed in healthy controls, possibly reflecting increased tissue uptake or higher metabolic demand, and suggesting a potential need for supraphysiological dosing strategies.

To allow comparison between treatments, serum CoQ10 concentrations were normalized for cumulative dose exposure, allowing a dose-adjusted comparison between formulations. Dose-normalized CoQ10 ratios (Cohen’s d = 7.39; median: 56.4-fold, range: 12.6–262.2) were higher under FSMP than with conventional ubidecarenone, indicating higher relative systemic exposure under the specific study conditions. These findings should not be interpreted as formal pharmacokinetic parameters, as standard metrics such as AUC, Cmax, and Tmax were not assessed. Rather, they provide an exploratory estimate of comparative exposure in a real-world setting. The cocoa butter-based lipid matrix of the FSMP may have contributed to enhanced intestinal uptake, consistent with previous evidence indicating improved absorption of lipophilic compounds with lipid-based formulations [28].

Substantial inter-individual variability was observed, likely reflecting differences in baseline treatment duration (12–240 months), individual absorption capacity, disease-related metabolic demands, and the transitional state following the formulation switch. Previous literature indicates that CoQ10 distribution to mitochondrial compartments may require prolonged exposure, potentially contributing to heterogeneous short-term responses [26,29].

In this context, multi-omics approaches may provide additional resolutions for interpreting such variability in PMD. By integrating genomic, transcriptomic, proteomic, and metabolomic data, these approaches can complement single circulating biomarkers and improve the characterization of patient-specific metabolic and molecular profiles. This is particularly relevant in light of the dissociation observed in the present study between circulating CoQ10, mitochondrial stress markers, and functional outcomes, suggesting that single-level biochemical measurements may not fully capture tissue-level bioenergetic responses. Multi-omics integration may therefore support more refined patient stratification and mechanistic interpretation of heterogeneous response patterns in future studies [30].

Despite the lipophilic nature of CoQ10, no significant association was observed between BMI and circulating CoQ10 levels or dose-normalized exposure parameters in patients, who were predominantly of normal weight (70%). This likely reflects the limited BMI variability within the cohort. In healthy controls, for whom only baseline CoQ10 was available, BMI was not significantly associated with circulating CoQ10 levels despite a wider distribution of normal weight, overweight, and obese individuals (10/7/3, respectively). By contrast, studies in populations with higher adiposity have reported lower plasma CoQ10 levels in obese subjects, suggesting that body composition may influence CoQ10 homeostasis mainly in populations with marked adiposity [31]. These observations were consistent with sensitivity analysis, which confirmed that between-group differences in CoQ10 levels remained significant after adjustment for BMI, whereas BMI was not independently associated with circulating CoQ10 concentrations. Overall, in our cohort, systemic CoQ10 availability may be influenced by formulation characteristics, individual lipid metabolism, and disease-related factors, than by body composition.

Circulating FGF21 and GDF15 levels, recognized biomarkers of mitochondrial diseases [32,33], did not change significantly over the 30-day observation period. Effective comparisons across studies remain challenging due to differences in study design, supplementation regimens (CoQ10 formulations, alone or in combination), sample size, and patient characteristics. Nonetheless, these findings align with previous studies showing persistently elevated FGF21 and GDF15 in patients with PMM, even after prolonged supplementation [33].

Given the multifactorial involvement of oxidative stress in PMD, biomarkers of oxidative damage as well as antioxidant capacity were evaluated. While AOPP and t-SH levels remained stable, FRAP levels increased significantly after FSMP supplementation. However, it should be considered that all oxidative stress parameters were within the ranges observed in a comparable control setting (FRAP ≥ 0.7 mmol/L, t-SH ≥ 0.4 mmol/L, AOPP ≤ 200 mmol/µL [34,35]), independently from CoQ10 formulation.

These findings suggest that the antioxidant effects of CoQ10 may primarily reflect enhanced ROS scavenging and support of endogenous antioxidant defenses [22]. Moreover, the lack of changes in oxidative damage and circulating mitochondrial stress markers may also be related to the short duration of the intervention or residual effects from prior supplementation.

Clinical outcomes, including fatigue (FSS) and physical performance (5xSST), did not show significant short-term changes following the 30-day intervention. Given the small sample size and limited follow-up, this study was not powered to detect clinically meaningful changes in functional outcomes. Therefore, no conclusions regarding clinical efficacy can be drawn. Previous studies in mitochondrial disorders have reported variable functional responses to CoQ10 supplementation depending on disease context, duration, and patient characteristics [36,37].

These observations support the plausibility of FSMP-mediated benefits while emphasizing the need for long-term studies to determine whether early trends translate into clinically improved outcomes. However, given the differences with literature evidence, no conclusions on clinical efficacy can be drawn.

Exploratory subgroup analyses suggested a phenotype-dependent response pattern following the formulation switch. Patients with point mutation-associated multisystem phenotypes showed increased circulating CoQ10, whereas those with predominantly myopathic phenotypes exhibited reduced serum levels, accompanied by numerically favorable trends in functional measures. These findings indicate a possible dissociation between circulating CoQ10 concentrations and clinical functional outcomes, suggesting that serum levels alone may not directly reflect tissue-level biological response. One interpretation is that circulating CoQ10 reflects a dynamic balance between absorption, distribution, and cellular utilization rather than systemic availability alone. In this context, lower circulating levels could potentially reflect increased tissue uptake, whereas higher levels may indicate more limited utilization. However, this interpretation remains speculative and should be interpreted cautiously given the exploratory nature of the analysis.

Responder analyses further illustrated the marked inter-individual variability. In the absence of validated minimal clinically important difference (MCID) thresholds for mitochondrial diseases, all criteria were considered exploratory. For FSS, 60%, 40%, and 30% of patients achieved reductions of ≥2, ≥3, and ≥4 points, respectively, while 20% of patients showed a >2 s improvement in 5×SST. These thresholds should be interpreted cautiously, as they are derived from other clinical contexts and are not disease-specific [38,39].

Overall, these observations underscore the limitations of relying on circulating CoQ10 as a standalone surrogate endpoint, particularly in tissues with high energetic demand such as skeletal muscle, highlighting the need for integrated biomarker approaches in future studies.

Several methodological limitations should be considered. The cohort was small and genetically heterogeneous, reflecting real-world complexity of PMD but limiting statistical power. The 30-day intervention period provides insight into early biochemical responses but is insufficient to evaluate longer-term functional outcomes. In addition, the 5-day washout period may not have fully eliminated residual effects of previous ubidecarenone supplementation, given the prolonged tissue persistence of CoQ10. Consequently, a carryover effect from previous supplementation cannot be excluded and should be considered when interpreting comparative exposure estimates between formulations. Furthermore, dose-normalized serum CoQ10 parameters–although informative and useful for estimating systemic exposure–do not replace formal pharmacokinetic profiling and should not be interpreted as such. Circulating biomarkers may also not fully reflect tissue-level redox and mitochondrial function. Collectively, these limitations underscore the exploratory nature of this study and support the need for larger, controlled studies with longer follow-up and formal pharmacokinetic assessment to better define the clinical and biochemical relevance of high-bioavailability CoQ10 formulations.

## 4. Materials and Methods

An open-label, single-center, feasibility trial evaluating Q250^®^ (Laboratori Aliveda S.r.l., Crespina Lorenzana, Pisa, Italy), a food for special medical purposes (FSMP), was conducted between August and November 2024 at a tertiary referral center for mitochondrial diseases in Italy.

### 4.1. Inclusion and Exclusion Criteria

Participants were eligible for inclusion if they met all of the following criteria:

- Age ≥ 18 years;
- Genetically confirmed diagnosis of primary mitochondrial disease (PMD);
- Ongoing treatment with ubidecarenone for at least 30 consecutive days prior to enrollment;
- Ability to independently perform the 5-times sit-to-stand test (5xSST);
- Provision of written informed consent.

Patients were excluded if they met any of the following criteria:

- Diagnosis of primary CoQ10 deficiency (PCoQ10D);
- Pregnancy or breastfeeding;
- Known intolerance to any component of the investigational product;
- Inability to independently perform the 5xSST;
- Participation in an interventional clinical trial at the time of screening.

### 4.2. Outcome Measures

Primary outcomes included changes in serum levels of CoQ10, FGF21, and GDF15 biomarkers.

In addition, complementary biomarkers reflecting protein peroxidation (advanced oxidation protein products, AOPP), and non-enzymatic antioxidant activity (ferric reducing antioxidant power, FRAP; total thiols, t-SH) were assessed.

Secondary clinical outcomes included functional performance assessed by 5xSST and fatigue severity measured by fatigue severity scale (FSS).

### 4.3. Study Protocol

Baseline assessments (T0) were performed while patients were still receiving conventional CoQ10 supplementation, thereby providing baseline clinical and biochemical measures. After T0, all patients underwent a 5-day washout from ubidecarenone. FSMP administration commenced immediately after the washout, and follow-up assessment (T1) was conducted 30 days after initiation of FSMP supplementation. FSMP was administered once daily (250 mg CoQ10) or twice daily (500 mg CoQ10), according to each patient’s prior CoQ10 regimen (Figure 5). For baseline comparisons, data from an age- and sex-matched historical cohort of healthy controls were used. Healthy controls had no history of mitochondrial, neurodegenerative, metabolic, or other disorders and were not taking supplements or other medications known to affect mitochondrial homeostasis and oxidative stress parameters. Blood samples and biomarker measurements in this cohort were performed using the same protocols as those applied to the patient cohort.

### 4.4. Investigational Product

The study product was an FSMP containing high-dose coenzyme Q10 (Q250^®^). The composition of each capsule is detailed as follows: coenzyme Q10 (ubiquinone, 250 mg), maltodextrin (bulking agent), hydroxypropyl methylcellulose (capsule shell), cocoa butter, and anti-caking agents (silicon dioxide, magnesium salts of fatty acids).

### 4.5. Biochemical Marker Assessment

All samples for each biomarker were assayed in duplicate using a microplate reader (Infinite M Nano, Tecan, Männedorf, Switzerland). Analyses were performed by a blinded operator, as detailed below.

#### 4.5.1. CoQ10 Assay

Serum CoQ10 levels were determined using a commercial human CoQ10 ELISA kit (Cusabio, Wuhan, China) according to the manufacturer’s instructions. Briefly, 50 µL of diluted serum (1:100) and 50 µL of horseradish peroxidase (HRP)-conjugated streptavidin were added to wells pre-coated with a monoclonal antibody specific for CoQ10. Plates were incubated at 37 °C for 40 min. After washing, 90 µL of TMB substrate was added and incubated for 20 min at 37 °C in the dark. The reaction was stopped by adding 50 µL of stop solution, and absorbance was measured at 450 nm with wavelength correction at 540 nm. CoQ10 concentrations were calculated from a standard curve and expressed in ng/mL. The assay detection range was 3.12–50 ng/mL, with a sensitivity of 1.56 ng/mL. Intra- and inter-assay coefficients of variation were <8% and <10%, respectively. All samples were measured within the assay’s dynamic range after appropriate dilution, and concentrations were corrected for the corresponding dilution factor. Consequently, final serum values may exceed the nominal assay range, which applies solely to the diluted samples used for interpolation.

#### 4.5.2. FGF21 Assay

Serum levels of FGF21 were quantified using a human FGF21 ELISA kit (Invitrogen, Thermo Fisher Scientific, Segrate, Italy), following the manufacturer’s instructions. Diluted samples (100 µL) were incubated in pre-coated wells with FGF21-specific antibodies for 2.5 h at room temperature. After washing, 100 µL of biotinylated detection antibody was added and incubated for 60 min, followed by 100 µL HRP-conjugated streptavidin for 45 min. TMB substrate (100 µL) was added for 30 min in the dark. Then the reaction was stopped with 50 µL of stop solution. Absorbance was measured at 450 nm and FGF21 concentrations were calculated from a standard curve and expressed in pg/mL.

#### 4.5.3. GDF15 Assay

Serum GDF15 levels were determined using a human GDF15 ELISA kit (R&D Systems, Minneapolis, MN, USA), following the manufacturer’s instructions. Fifty microliters of diluted serum (1:4) were added to wells pre-coated with GDF15-specific antibodies and incubated for 2 h at room temperature on a shaker. After washing, 200 µL of human GDF15 conjugate was added for 60 min, followed by 200 µL of substrate solution for 30 min. The reaction was stopped with 50 µL stop solution, and absorbance was measured at 450 nm with wavelength correction at 540 nm. Concentrations were calculated from a standard curve and expressed in pg/mL.

#### 4.5.4. FRAP Evaluation

FRAP assay was performed to evaluate non-enzymatic antioxidant capacity. Pre-warmed (37 °C) FRAP reagent, consisting of sodium-acetate, 2,4,6-tripyridyl-s-triazine in hydrochloric acid, and ferric chloride (Sigma-Aldrich, Milano, Italy) in a 10:1:1 ratio, was mixed with 8 µL of plasma. Absorbance was measured at 620 nm after 4 min. A calibration curve was prepared using iron sulfate in hydrochloric acid and results were expressed in mmol/L [40].

#### 4.5.5. t-SH Evaluation

T-SH were quantified following Hu [41]. Plasma was mixed into Tris–EDTA solution, 5,5-dithiobis-2-nitrobenzoic acid (Sigma-Aldrich, Milano, Italy), and absolute methanol (Honeywell, Offenbach, Germany), and incubated at room temperature for 20 min. Samples were centrifuged at 3000× *g* for 10 min, and the absorbance of the supernatant was measured at 405 nm. Values were expressed in mmol/L of -SH groups.

#### 4.5.6. AOPP Evaluation

AOPP were measured according to Witko-Sarsat et al. [42]. Briefly, plasma (30 µL) was mixed with 170 µL PBS, 20 µL acetic acid, and 10 µL potassium iodide (Sigma-Aldrich, Milano, Italy). Absorbance was read spectrophotometrically at 340 nm using a microplate reader and compared with a chloramine T standard solution (Sigma-Aldrich, Milano, Italy). Data were expressed as nmol/mL of chloramine equivalents.

### 4.6. Statistical Analysis

Statistical analyses were performed using GraphPad Prism version 5.0 (GraphPad Software, San Diego, CA, USA), Jamovi (version 2.6.44), and Microsoft Excel for Windows. Categorical variables are presented as frequencies (*n*) and percentages (%), while continuous variables are reported as mean ± standard deviation (SD). The Shapiro–Wilk test was used to assess normality of data distribution.

For within-subject comparisons between baseline (T0) and follow-up (T1), parametric paired *t*-tests were applied for normally distributed data, whereas non-parametric Wilcoxon matched-pairs signed-rank tests were used for non-normally distributed data. Between-group comparisons (mitochondrial disease patients vs. healthy controls) were performed using the Mann–Whitney U test. Associations between continuous variables were assessed using Pearson’s correlation for normally distributed data and Sperman’s rank correlation for non-normally distributed data.

Given the substantial inter-individual variability in administered doses and treatment duration, serum CoQ10 concentrations were normalized for cumulative dose exposure. A dose-normalized ratio (Ratio_norm) was calculated to enable quantitative comparison between formulations (Equation (1)). Based on Ratio_norm, a relative dose-normalized exposure ratio (Q250/Ubidecarenone) was calculated to compare systemic CoQ10 exposure between formulations under real-world conditions (Equation (2)).

Ratio_norm = serum CoQ10 concentration/cumulative dose(1)

Relative dose-normalized exposure ratio = Ratio_norm Q250/Ratio_norm Ubidecarenone(2)

Effect size was quantified using Cohen’s d (paired), calculated on CoQ10 Ratio_norm values. Sensitivity analyses were performed using a general linear model (ANCOVA), with CoQ10 concentrations as the dependent variable, group (patients vs. healthy controls) as fixed factor, and BMI as covariate.

Given the exploratory nature of the study, phenotype-based subgroup analyses were performed to describe inter-individual variability. The analyses were descriptive and no formal statistical comparisons between subgroups were conducted.

All statistical tests were two-tailed, with a 95% confidence interval, and statistical significance defined as *p* < 0.05.

## 5. Conclusions

This 30-day, single-arm, pilot feasibility study indicates that the Q250^®^ formulation, administered after conventional ubidecarenone, was generally well-tolerated during the study period and was associated with higher dose-normalized systemic CoQ10 exposure. The cocoa butter-based lipid matrix may contribute to the higher relative absorption efficiency and reduced inter-individual variability, without worsening mitochondrial and oxidative stress biomarkers.

Although no significant short-term changes in fatigue or physical performance were observed, exploratory subgroup analyses revealed a dissociation between circulating CoQ10 levels and functional antioxidant outcomes, particularly in patients with predominantly myopathic phenotypes. These findings suggest that serum CoQ10 concentrations alone may not adequately reflect tissue-level mitochondrial response in high-energy-demand tissues such as skeletal muscle.

Overall, this study supports the feasibility of CoQ10 formulations associated with more consistent and sustained systemic CoQ10 exposure while simplifying supplementation regimens in PMD. However, the exploratory nature of the findings and the limited sample size preclude conclusions regarding clinical efficacy.

Future controlled studies with larger cohorts, longer follow-up, tissue-level biomarkers, and disease-specific outcome measures will be necessary to clarify the relationship between circulating CoQ10, tissue bioavailability, and long-term clinical benefit in mitochondrial disorders.

## Figures and Tables

**Table 1:** Baseline general features of patients.

ID	Gender	Age	BMI	Phenotype	Age at Onset	Disease Duration (Months)	Ubidec Treatment (Months)	Ubidec (mg/day)	FSMP (mg/day)	Ref.
AL01	M	56	19.4	MELAS	47	108	108	500	500	
AL02	F	62	23.8	PMM ^1^ (MiMy spectrum without PEO)	54	106	106	200	250	[18]
AL03	M	42	21.4	MELAS	38	51	45	200	250	
AL04	M	46	14.3	MIDD	68	68	68	600	500	
AL05	F	53	19.3	NARP	26	324	12	200	250	
AL06	M	50	29.6	PMM ^1^ (PEO spectrum)	16	408	180	250	250	[18]
AL07	F	60	20.0	PMM ^1^ (PEO spectrum)	11	588	240	300	250	[18]
AL08	F	68	18.4	PMM ^1^ (PEO spectrum)	50	216	24	200	250	[18]
AL09	M	67	23.0	PMM ^1^ (MiMy spectrum without PEO)	60	72	44	200	250	[18]
AL10	F	51	20.9	KSS spectrum ^2^	20	372	60	400	500	[19]

^1^ PPM defined according to Mancuso et al. [18]; ^2^ KSS spectrum defined according to Mancuso et al. [19]. Abbreviations: M, male; F, female; Ubidec, ubidecarenone; MELAS, mitochondrial encephalomyopathy, lactic acidosis, stroke-like episodes; PMM, primary mitochondrial myopathies; PEO, progressive external ophthalmoplegia; MiMy, mitochondrial myopathy; KSS, Kearns–Sayre syndrome; MIDD, maternally inherited diabetes and deafness; NARP, neuropathy, ataxia, and retinitis pigmentosa.

**Table 2:** Exploratory individual longitudinal responses by mitochondrial disease subgroup.

ID	Subgroup	Formulation Switch (mg)	CoQ10 T0	CoQ10 T1	ΔCoQ10	Δ5xSST	ΔFSS	ΔFRAP
AL01	Point mutation/ multisystem	Unchanged	703.2	442.1	−261.1	+1.67	−2	+0.048
AL02	Myopathy	Increased	634.5	448.6	−185.9	−1.11	−8	+0.523
AL03	Point mutation/ multisystem	Increased	357.4	399.6	+42.2	+2.53	+3	+0.295
AL04	Point mutation/ multisystem	Decreased	435	725.9	+290.9	+5.80	+17	+0.015
AL05	Point mutation/ multisystem	Increased	233.5	411.3	+177.8	−5.93	−2	+0.222
AL06	Myopathy	Unchanged	389.3	470.5	+81.2	−0.4	−15	+0.153
AL07	Myopathy	Decreased	540.5	492	−48.5	−1.68	−3	+0.152
AL08	Myopathy	Increased	621.5	406.7	−214.8	+1.50	−11	+0.339
AL09	Myopathy	Increased	373.6	394.1	+20.5	−1.81	+8	−0.078
AL10	Myopathy	Increased	736.6	809.6	+37.0	−2.43	+2	+0.161

Subgroups were defined based on predominant clinical phenotype. The “point mutation with multisystem involvement” subgroup includes MELAS, MIDD, and NARP, characterized by predominant multisystem and central nervous system involvement. The “myopathy” subgroup includes mitochondrial myopathy phenotypes with and without PEO, single mtDNA deletion-associated conditions, and KSS spectrum, all characterized by predominant skeletal muscle involvement. Formulation switch refers to the change in daily CoQ10 dose following the transition from ubidecarenone to the FSMP.

**Table 3:** Phenotype-stratified changes in biochemical and functional outcomes.

Group	Subgroup	N	ΔCoQ10	Δ5xSST	ΔFSS	ΔFRAP
Phenotype	Point mutation/Multisystem	4	+62.5	+1.02	+4.0	+0.154
	Myopathy/Deletion	6	−51.8	−0.99	−4.5	+0.208

## Data Availability

The datasets generated during and/or analyzed during the current study are available from the corresponding author on reasonable request.

## Supplementary Materials

The following supporting information can be downloaded at: https://www.mdpi.com/article/10.3390/ijms27115127/s1.

## Abbreviations

The following abbreviations are used in this manuscript:

5xSST	5-times sit-to-stand test
AOPP	Advanced oxidation protein products
ATP	Adenosine triphosphate
CoQ10	Coenzyme Q10
EMA	European medicines agency
FGF21	Fibroblast growth factor 21
FRAP	Ferric reducing antioxidant power
FSMP	Food for special medical purposes
FSS	Fatigue severity scale
GDF15	Growth differentiation factor 15
KSS	Kearns–Sayre syndrome
LHON	Leber hereditary optic neuropathy
MELAS	Mitochondrial encephalomyopathy, lactic acidosis, stroke-like episodes
MIDD	Maternally inherited diabetes and deafness
MiMy	Mitochondrial myopathy
MCID	Minimal clinically important difference
mtDNA	Mitochondrial DNA
NARP	Neuropathy, ataxia, and retinitis pigmentosa
nDNA	Nuclear DNA
OXPHOS	Oxidative phosphorylation
PCoQ10D	Primary CoQ10 deficiency
PEO	Progressive external ophthalmoplegia
PMD	Primary mitochondrial diseases
PMM	Primary mitochondrial myopathies
t-SH	Total sulfhydryl groups
Ubidec	Ubidecarenone
