# Supplementary material for: Evaluating a Coenzyme Q10-Based Food for Special Medical Purpose, for Mitochondrial Diseases Management: An Open-Label, Pilot Trial
Source: Int J Mol Sci. 2026 Jun 5;27(11):5127. doi: 10.3390/ijms27115127 (PMC13257338; doi:10.3390/ijms27115127)
Supplement: Supplementary file 1 [file ijms-27-05127-s001.zip › ijms-4308763-supplementary.pdf]

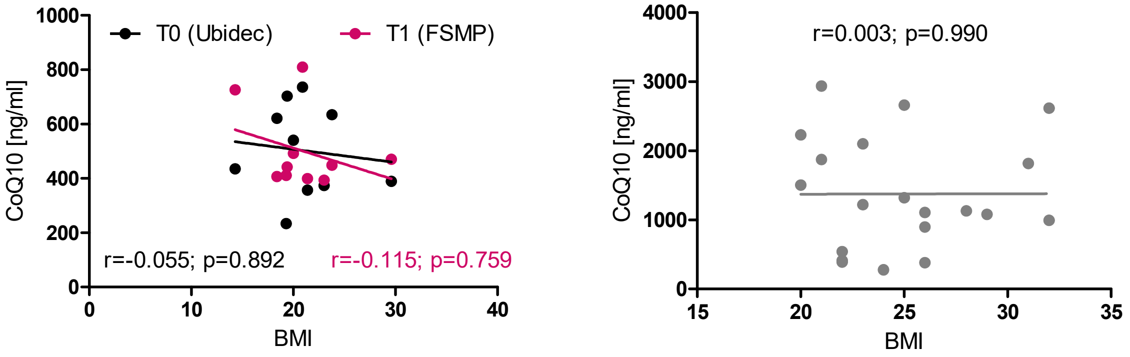

(**A**) (**B**)

Figure S1. Correlation analysis of CoQ10 levels with BMI. (**A**) PMD patients at T0 and T1; (**B**) Healthy controls.

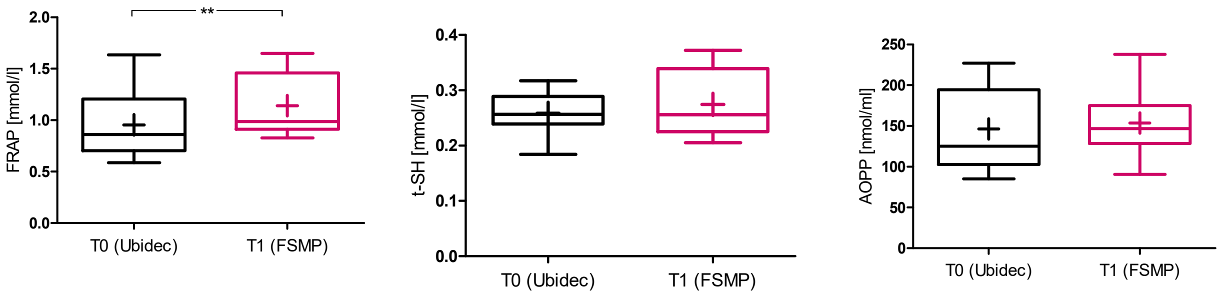

(**A**) (**B**) (**C**)

Figure S2. Oxidative stress biomarkers at T0 and T1. (**A**) Non-enzymatic antioxidant capacity (FRAP); (**B**) Total thiol groups (t-SH); (**C**) Oxidative damage to proteins (AOPP). T0: conventional ubidecarenone; T1: FSMP. ** p<0.01.

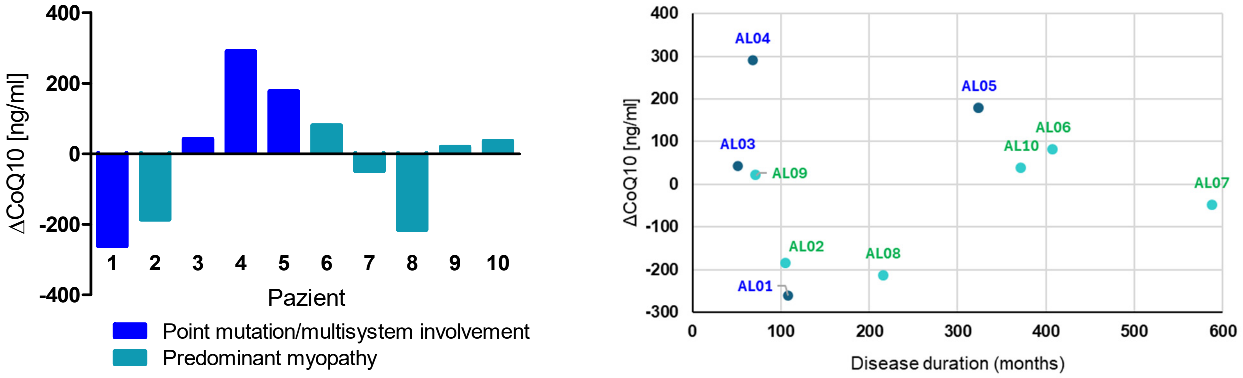

(**A**) (**B**)

Figure S3. Individual variation in serum CoQ10 concentrations (ΔCoQ10) following formulation switch. (**A**) ΔCoQ10 for each patient; (**B**) ΔCoQ10 plotted against disease duration (months). Blue bars/dots indicate patients with point mutation and multisystem involvement; green bars/dots indicate patients with predominant myopathy.

Table S1. Patient characteristics and specific ubidecarenone formulations used prior to FSMP switch.

ID	mtDNA mutation	Phenotype	Muscle manifestations	Brand (Ubidec formulation	Type	Manufacturer
AL01	m.3243 A>G	MELAS	Mild exercise intolerance, mild ptosis	Ubimaior®	Rigid capsule (50 mg)	Chiesi Italia s.p.a.
AL02	m.5835 G>A	PMM (MiMy)	Muscle weakness, exercise intolerance	Q10 Gold®	Capsule (100 mg)	PharmaNord s.r.l.
AL03	m.3243 A>G	MELAS	Mild exercise intolerance	Q200®	Capsule (200 mg)	Laboratori Aliveda s.r.l.
AL04	m.3243 A>G	MIDD	-	Q200®	Capsule (200 mg)	Laboratori Aliveda s.r.l.
AL05	m.8993 T>G	NARP	Ophthalmoparesis	Q200®	Capsule (200 mg)	Laboratori Aliveda s.r.l.
AL06	single deletion	PMM (PEO)	Ptosis, ophthalmoparesis	Ubimaior®	Rigid capsule (50 mg)	Chiesi Italia s.p.a.
AL07	single deletion	PMM (PEO)	Ptosis, ophthalmoparesis, muscle weakness, bulbar weakness, respiratory muscle involvement	Q10 Gold®	Capsule (100 mg)	PharmaNord s.r.l.
AL08	single deletion	PMM (PEO)	Ptosis, ophthalmoparesis, bulbar weakness,	Ubimaior®	Rigid capsule (50 mg)	Chiesi Italia s.p.a.
AL09	m.8344 A>G	PMM (MiMy)	Mild ptosis, exercise intolerance	Q10 Gold®	Capsule (100 mg)	PharmaNord s.r.l.
AL10	single deletion	KSS spectrum	Ptosis, ophthalmoparesis, respiratory muscle involvement	Q10 Gold®	Capsule (100 mg)	PharmaNord s.r.l.

Table S2. Correlation analysis of CoQ10 levels (dose-normalized exposure parameters) with BMI in PMD patients.

BMI–CoQ10	Formulation	Correlation coefficient (r)	p-value
Normalized ratio	Ubidecarenone (T0)	-0.212	0.560
	FSMP (T1)	0.370	0.296
Relative dose-normalized exposure ratio	FSMP/Ubidecarenone	0.212	0.560

No significant correlations were found (all p>0.05).

Table S3. General linear model (ANCOVA) for CoQ10 levels adjusted for BMI.

				95% Confidence Intervals					
Names	Effect	Estimate	SE	Lower	Upper	β	df	t	p-value
(Intercept)	(Intercept)	938.267	136.256	658.692	1217.842	-0.185	27	6.886	<.001
Group	Patient - HC	-877.958	299.281	-1492.032	-263.883	-1.111	27	-2.934	0.007
BMI	BMI	-1.242	33.733	-70.457	67.973	-0.007	27	-0.037	0.971

The model included group (HC vs. patients) as fixed factor and BMI as covariate. Confidence intervals (95%) are reported for parameter estimates. β values represent standardized coefficients. HC, healthy control.

Table S4. Individual percentage changes (Δ%) in serum CoQ10 levels, 5xSST, and FSS.

ID	Subgroup	Δ%CoQ10	Δ%5xSST	Δ%FSS
AL01	Point mutation/ multisystem	-37.0	13.5	-4.2
AL02	Myopathy	-29.0	-5.6	-17.0
AL03	Point mutation/ multisystem	11.0	30.5	16.7
AL04	Point mutation/ multisystem	66.0	31.2	42.5
AL05	Point mutation/ multisystem	76.0	-24.5	-4.1
AL06	Myopathy	20.0	-3.8	-39.5
AL07	Myopathy	-9.0	-10.0	-5.1
AL08	Myopathy	-34.0	8.5	-19.6
AL09	Myopathy	5.5	-13.3	18.2
AL10	Myopathy	9.5	-20.9	5.7

Table S5. Exploratory responder analysis for functional outcomes (FSS and 5xSST).

Endpoint	Response threshold	Responders (%)
FSS	Reduction ≥2 points	60
	Reduction ≥3 points	40
	Reduction ≥4 points	30
5xSST	Reduction ≥ 2 seconds	20
